# Continuous suturing with two anterior layers reduces post-operative complications and hospitalization time in pancreaticoenterostomy

**DOI:** 10.1186/s12876-016-0482-8

**Published:** 2016-07-11

**Authors:** Guoliang Yao, Yonggang Fan, Jingming Zhai

**Affiliations:** Department of General Surgery, The First Affiliated Hospital of Henan University of Science and Technology, 24 Jinghua Road, Luoyang, 471003 People’s Republic of China

**Keywords:** Pancreaticoenterostomy, Pancreaticoduodenectomy, Pancreatic leakage, Continuous mattress suturing

## Abstract

**Background:**

Most complications after pancreaticoduodenectomy (PD) were relation to pancreaticoenterostomy. We improved a new method of pancreaticoenterostomy that included the continuous suturing of the jejunum and the stump of the pancreas end-to-side with one layer posteriorly and two layers anteriorly. To evaluate the safety and efficiency of this new method, we introduced this retrospectively compared trial.

**Methods:**

We compared 45 patients who had undergone pancreaticoduodenectomy with either the regular interrupted suturing method or the new continuous mattress suturing method in our hospital from September 2011 to March 2014.

**Results:**

Although the total operation times were not reduced, the suturing time for the pancreaticoenterostomies in the continuous suture group (11.3 ± 1.8 min) was greatly reduced compared with that for the interrupted suture group (14.1 ± 2.9 min, *p* = 0.045). Importantly, the continuous mattress suturing method significantly decreased short-term post-operative complications, including pancreatic leakage (*p* = 0.042). Furthermore, shorter hospitalization times were observed in the continuous mattress suture group (12.3 ± 5.0 d) than in the interrupted suture group (24.2 ± 11.6 d, *p* = 0.000).

**Conclusions:**

Continuous mattress suturing is a safe and effective pancreaticoenterostomy method that leads to reduced complications and hospitalization times.

## Background

Pancreaticoduodenectomy (PD) has been rapidly developed since it was first introduced. PD is used not only for peri-ampullary malignant tumors but also for certain benign pancreatic disorders. PD is a relatively safe surgery because its recent mortality rate has been reported to be only approximately 3–5 % [1–3]. However, the post-operative complications of PD have not been greatly reduced [4, 5]. Several modifications have been used to produce better outcomes, but they are complicated and time consuming [6]. Thus far, there is still no worldwide-accepted procedure to reduce complications. Here, we introduce a safe and effective procedure to reduce the complications and provided better outcomes.

## Methods

### Patient characteristics

We retrospectively analyzed all PDs performed because of peri-ampullary tumors in our hospital between September 2011 and March 2014. Patients with diffused metastases in the abdomen were excluded. Patients with severe diseases in other systems were also excluded because of their poor tolerances. From September 2011 to August 2013, 29 patients underwent PD with interrupted suturing. Because two patients died after their second laparotomies in August 2013 because of hemorrhaging secondary to pancreatic leakage, we modified the pancreaticoenterostomy procedure to include a new method of continuous suturing. By March 2014, 16 patients had undergone PD with continuous suturing by the same surgeon, who had more than 10 years’ experience with PD. The patient information, including basic characteristics, such as age and gender, and operation-related characteristics, such as the operation time, pancreaticoenterostomy time, hospitalization time, blood lost during the operation, and complications including pancreatic leakage and mortality were analyzed. According to the International Study Group for Pancreatic Fistula, pancreatic leakage was defined as drainage of any volume on or after postoperation d 3 with an amylase content greater than 3-fold the upper normal serum value.

### Operation procedure

**Fig. 1:**
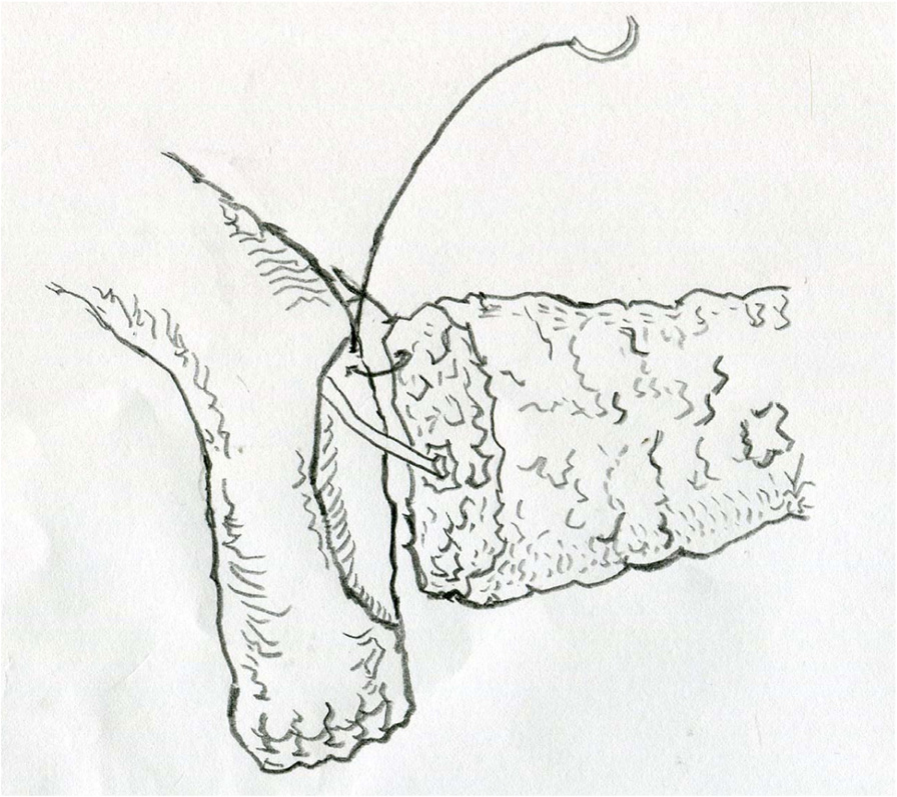
The first stitch was located at the upper edge of the pancreas. It’s beginning from the outside to the inside of the pancreas, and then from the inside to the outside of the jejunum and then a knot was tied outside

**Fig. 2:**
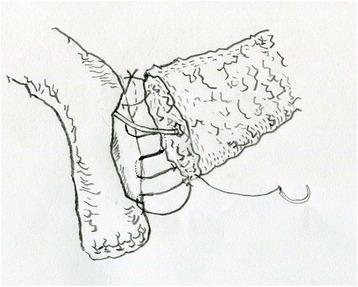
The posterior of the anastomosis was from upper edge to the lower edge. All the stitches were from the outside to the inside of the jejunum and then transfix the pancreas, and then the stump of pancreas was sutured with jejunum from the inside to the outside. The procedure was repeated until the very lower edge of the pancreas

**Fig. 3:**
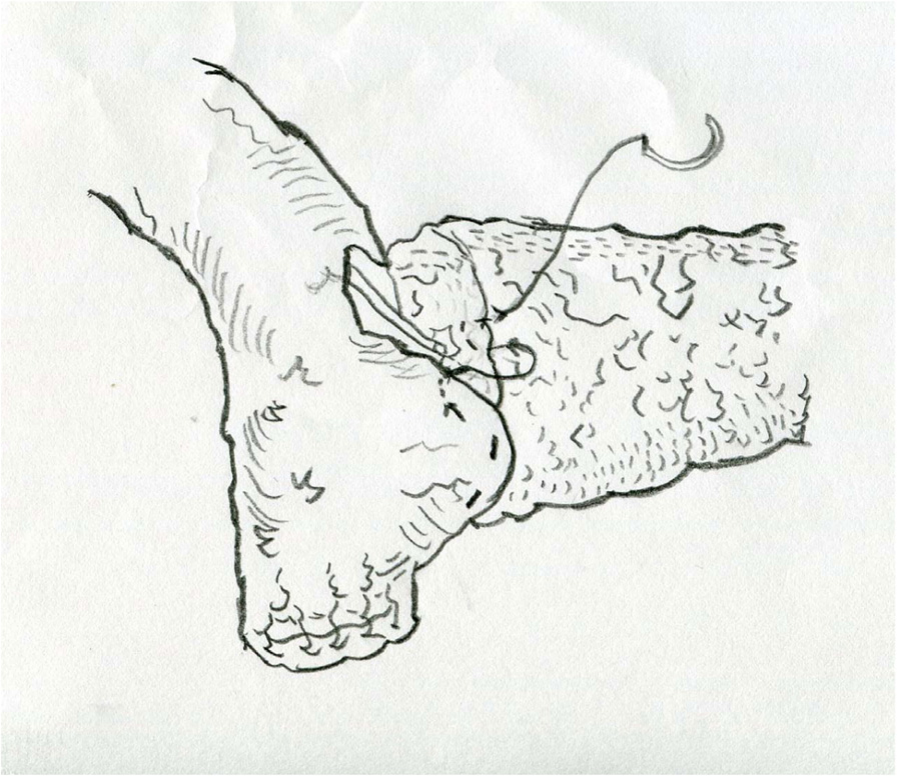
The anterior side of the anastomosis was continuously sutured just like the posterior side. All the stitches were from the outside to the inside of the jejunum and then transfix the pancreas. Then the stump of pancreas was sutured with jejunum from the inside to the outside. The procedure was repeated until the very upper edge of the pancreas. A knot was tied at the very end of the suture shown in Fig. 4 blow

**Fig. 4:**
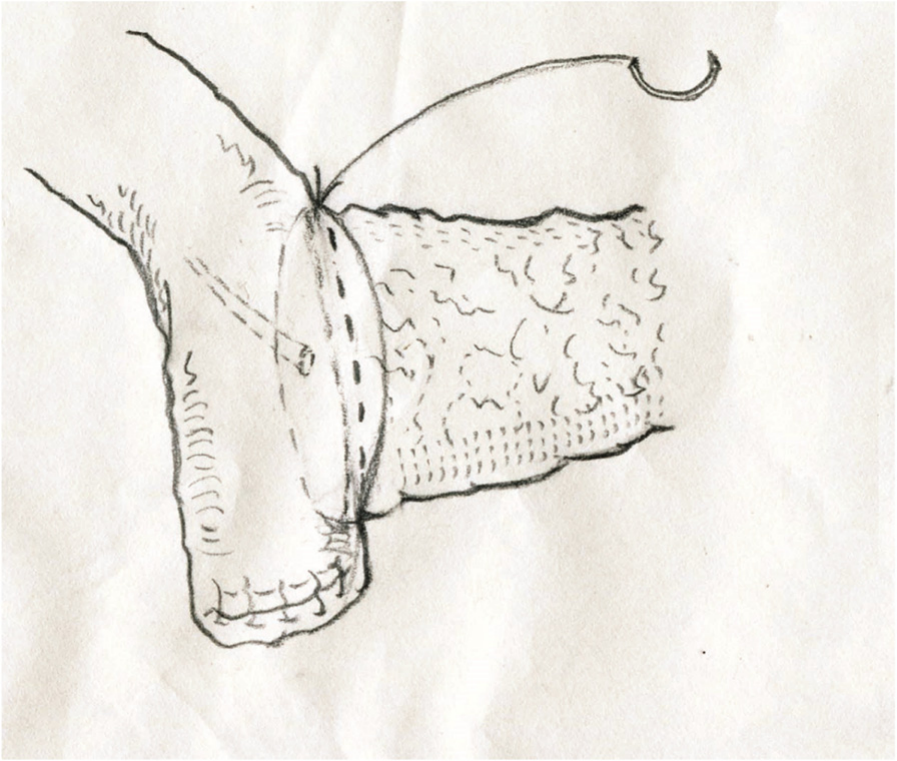
A knot was tied at the very upper edge of the pancreas and the first layer suture was finished

**Fig. 5:**
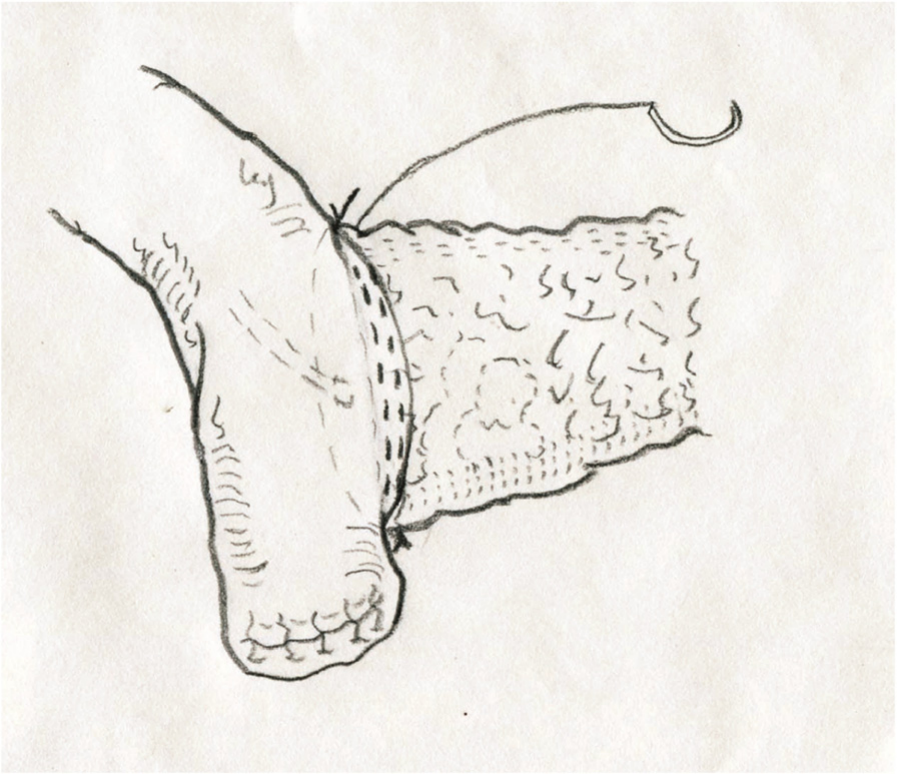
The second layer suture of the anterior side was location at the very edge of the jejunum just like the first layer. After this suture, the whole anastomosis was finished

The patients were sufficiently physiologically and psychologically prepared before the operations. During the operations, the transfixations of the upper and lower edges of the stump of the pancreas were emphasized to decrease blood loss before the transection of the pancreas. A pancreatic duct stent was used to the fix the stump of pancreas for at least 15 cm to drain the pancreatic jaundice to the distal end of the jejunum and was placed at least 10 cm away from the anastomosis of the cholangioenterostomy. The stump of the jejunum was pulled to the stump of the pancreas behind the transverse mesocolon without tension. The stump of the pancreas was invaginated into the jejunum by at least 2–3 cm and fixed with 3-0 polypropylene suture (Prolene, Ethicon). The difference between the continuous suturing and interrupted suturing was limited to the procedure of suturing the pancreaticoenterostomy. The interrupted suturing involved one layer of discontinuous sutures with distances of 2–3 mm between each pair of stitches. The continuous suturing involved one layer posteriorly and two layers anteriorly. A 3-0 polypropylene suture was used to complete the suturing from the very upper edge of the pancreas to the lower edge through the posterior edge of the pancreas, and the anterior suture was then completed with the suture. Finally, a knot was tied at the upper edge of the pancreas with the very end of the suture (Figs. 1, 2, 3 and 4). After the first-layer suture, a second-layer suture was applied from at the end edge of the lateral opening of the jejunum anteriorly from the very lower edge of the pancreas to the very upper edge (Fig. 5).

### Statistical analysis

SPSS 16.0 was used to analyze the data. The measurement data, including age, operation time, pancreaticoenterostomy time, blood loss, and hospitalization time, were compared with t tests. The numerical data, such as tumor location, pancreas texture, American Society of Anesthesiologists (ASA) classification, and complications, were compared with chi square tests. *P* < 0.05 was considered to be significant.

## Results

The basic characteristics of the two groups, including gender, age, carcinoma location, pancreas texture and ASA classification, are presented in Table 1. Two duodenal interstitialomas were found in the interrupted suture group, and one duodenal carcinoid was found in the continuous suture group. Advanced stage patients with portal vein or inferior vena cava invasion were excluded. There were no significant differences between the two groups in terms of age, gender, tumor location, degree of anemia, pancreatic texture, ASA score, blood loss or total operation time (Table 2). However, the pancreaticoenterostomy time in the continuous suture group was 11.3 ± 1.8 min, which was significantly shorter than the 14.1 ± 2.9 min observed in the interrupted suture group (*p* = 0.045). The hospitalization time was also significantly shorter for the continuous suture group (12.3 ± 5.0 d) than the interrupted suture group (24.2 ± 11.6 d, *p* = 0.000). Furthermore, the total complications were significantly decreased in the continuous suture group compared with the interrupted suture group (*p* = 0.042). There were three cases of death in the interrupted suture group and one case of death in the continuous suture group. Two out of these three deaths occurred after the second laparotomies, owing to hemorrhaging secondary to pancreatic leakage. The other death in the interrupted suture group was due to hemorrhaging without pancreatic leakage. The death in the continuous suture group occurred because of hemorrhaging secondary to pancreatic leakage without a second laparotomy. Regarding pancreatic leakage, according to the criteria of the International Study Group on Pancreatic Fistula (ISGPF), we defined leakage as a drain output of any measurable volume of fluid on or after postoperation day 3 with an amylase activity three times greater than that in the serum [4]. There were eight cases of pancreatic leakage in the interrupted suture group and two cases in the continuous suture group (Table 3). According to the ISGPF, one case was grade A, three cases were grade B and four cases were grade C in the interrupted suture group, whereas one case was grade B and one case was grade C in the continuous suture group (Table 3). However, neither the incidence (*p* = 0.585) nor the severity (*p* = 0.292) of pancreatic leakage was significantly different between groups. In the eight cases of pancreatic leakage in the interrupted suture group, two died after the second laparotomies, two exhibited bleeding secondary to leakage and were cured conservatively, and the other four were cured without other secondary injuries. In the continuous suture group, one case of pancreatic leakage died because of a secondary injury of a large hemorrhage, and the other case was cured conservatively.

**Table 1:** Basic clinic characteristics of the patients

Characteristics	Interrupt suturing (*N* = 29)	Continuous suturing (*N* = 16)	P
Gender (M/F)	18/11	11/5	0.752
Age (y)	67.3 ± 7.4	61.2 ± 6.2	0.482
Location			0.676
Jejunum	14^a^	5^b^	
Lower bile duct	4	4	
Ampulla	8	5	
Head of pancreas	3	2	
Anemia	86.3 ± 17.5	83.9 ± 18.3	0.793
ASA stage			0.901
I	5	2	
II	13	8	
III	11	6	
Pancreas texture			0.868
Hard	7	5	
Firm	17	7	
Soft	5	4	

^a^Including two cases of duodenal interstitialomas. One presented with melena, and the other was discovered via an upper digestive tract endoscopy examination for a non-specific abdominal distension syndrome

^b^Including a duodenal carcinoid that was discovered via an upper digestive tract endoscopy examination for abdominal distension and interrupted melena

**Table 2:** The operative characteristics of the patients

Characteristics	Interrupt suturing (*N* = 29)	Continuous suturing (*N* = 16)	P
Operation time (min)	260.8 ± 35.6	249.5 ± 31.7	0.731
Pancreaticoenterostomy time (min)	14.1 ± 2.9	11.3 ± 1.8	0.045
Blood lost (ml)	465.4 ± 72.3	426.1 ± 57.6	0.672
Hospitalization time (d)	24.2 ± 11.6	12.3 ± 5.0	0.000
Complications^a^			0.042
Death	3	1^b^	
Pancreatic leakage	8	2	
Bleeding	7	2	
Pneumonia	2	1	

^a^ The complications were varied. In the interrupted suture group, two of the three deaths were due to pancreatic leakage followed by severe hemorrhaging, as revealed by secondary laparotomy, and active bleeding occurred at the stomas of the pancreaticojejunostomies. The other death was due to hemorrhaging without pancreatic leakage. Two cases of bleeding that presented with post-operative blood drainage were cured conservatively and were secondary to pancreatic leakage. The remaining two cases of bleeding were also cured conservatively, and these cases presented with hematemesis and melena without pancreatic leakage. The remaining cases of pancreatic leakage were cured conservatively, and secondary injuries were not found

^b^ The death in the continuous group was also due to a large hemorrhage secondary to pancreatic leakage. The other case of pancreatic leakage was cured conservatively. The other case of bleeding in continuous group presented with melena and was cured conservatively. The case with pneumonia was cured by the time of discharge

**Table 3:** Pancreatic leakage classification according to the ISGPF

	Interrupt suturing (*N* = 29)	Continuous suturing (*N* = 16)	P
Pancreatic leakage	8(27.6 %)	2(12.5 %)	0.585
Severity classification			0.292
Grade A	1	0	
Grade B	4	1	
Grade C	3	1	

## Discussions

PD is the optimal choice for the peri-ampullary tumors [7]. Although the mortality after PD is low, the post-operative morbidity remains as high as 30–65 % [3, 8–13]. Pancreatic fistulae are the most serious postoperative complication and may cause a series of secondary injuries, and even death [14]. Many efforts have been made to reduce the occurrence of pancreatic fistulae. Baki Topal et al [7] have reported that pancreaticogastrostomy can reduce the clinical and biochemical pancreatic fistulae compared with pancreaticojejunostomy. However, pancreaticogastrostomy has no advantage in reducing the overall postoperative complications. Moreover, Bassi C et al [15] have reported contradictory results and have found no significant differences in pancreatic leakage between pancreaticogastrostomy and pancreaticojejunostomy. Pancreatic duct stent placement is a widely adopted improvement that may reduce pancreatic leakage, morbidity and mortality after PD [16–18]. However, stenting increases the operation cost. Additionally, Seung Eun Lee et al [19] have found that continuous stitching is more feasible and safe than interrupted stitching during the performance of duct-to-mucosa pancreaticojejunostomy. However, this modification is a complicated procedure with an extended operation time.

Here, we introduced a small modification that led to a substantial improvement in postoperative morbidity. We performed continuous stitching of the pancreaticojejunal anastomosis involving one layer posteriorly and two layers anteriorly rather than interrupt sutures. We used two layers anteriorly because two of the patients who died in the interrupt suture group had ulcers located at the anterior of the pancreaticojejunal anastomotic stoma with active bleeding. This enhanced anterior suturing with two layers is a very simple procedure that requires only a 3-0 polypropylene suture. We sutured the jejunum and the stump of pancreas from the very upper edge of the pancreas to the lower edge through the posterior edge of the pancreas and then continuously completed the anterior suture with the same suture. Finally, a knot was tied at the upper edge of pancreas with the very end of the suture. In the first layer, only two knots were needed. Moreover, the second anterior layer was also continuously completed. Continuous suturing has at least four advantages: First, a more even distribution of tension can be achieved between the pancreatic parenchyma and the jejunum [20]. Second, owing to the coiled spring effect, the continuous suturing method also provides a reduction in the likelihood of focal tissue ischemia, an increase in tensile strength, and a reduction of the risk of pancreaticojejunal rupture [20]. Third, continuous suturing reduces the anastomosis time. Finally, continuous suturing is technically easier and costs less [21].

Our results revealed a shortened pancreaticojejunostomy time due to the simple procedure involving the end-to-side invagination technique. This technique required only 11.3 ± 1.8 min to complete the anastomosis. Because of the advantages of the continuous suturing, fewer cases with severe complications and shorter hospitalization times were achieved. Although neither the incidence nor severity of pancreatic leakage were different between the two groups, our results revealed a trend toward a decline (27.6 % vs 12.5 %). This trend may explain the decline in the total complications, which led to shorter hospitalizations. As a preliminary investigation, our study included a relatively small number of patients, and this may have influenced the results.

## Conclusions

Continuous mattress suturing is a safe and effective pancreaticoenterostomy method that leads to reduced complications and hospitalization times.

## Abbreviations

ASA, American Society of Anesthesiologists; ISGPF, the International Study Group on Pancreatic Fistula; PD, pancreaticoduodenectomy
